# Bioactive glass versus Arginine dentifrices on the reduction of dentin permeability and acid tolerance

**DOI:** 10.1002/cre2.372

**Published:** 2020-12-06

**Authors:** Chantrakorn Champaiboon, Attawood Lertpimonchai, Kullanun Lertpimonchai

**Affiliations:** ^1^ Department of Periodontology, Faculty of Dentistry Chulalongkorn University Bangkok Thailand; ^2^ Center of Excellence in Periodontal Disease and Dental Implant Chulalongkorn University Bangkok 10330 Thailand

**Keywords:** acid tolerance, arginine, calcium sodium phosphosilicate, dentin hypersensitivity

## Abstract

**Objectives:**

To compare the efficacy of calcium sodium phosphosilicate (CSPS) and arginine dentifrices on dentin permeability and acid tolerance.

**Material and Methods:**

Sixty dentin discs were randomly assigned into 3 groups, then brushed for 1 min with CSPS, arginine, or fluoride (control) dentifrices. To test acid tolerance, each disc was soaked in 6% citric acid for 1 min. Dentin permeability was measured before, following brushing, and acid challenge. Ten discs per group were similarly treated and evaluated for tubule occlusion following a single dentifrice application, while other five discs per group were employed in an acid tolerance assay.

**Results:**

The percentage reduction in dentin permeability was 39.26%, 32.27%, and 21.71% in the arginine, CSPS, and control groups, respectively. The differences in dentin permeability reduction between the arginine and CSPS groups following brushing and acid challenge were not significant (*p* = 0.398 and *p* = 0.211, respectively). The arginine dentifrice demonstrated a significant reduction in permeability compared with the control (*p* = 0.011). In addition, the occlusion exhibited by the arginine and CSPS dentifrices was more resistant to acid challenge compared with that of the control (*p* < 0.001). From SEM analysis, dentinal tubule occlusion was observed after a single application in all groups. Some open dentinal tubules were detected in the test groups, while almost all of the orifices were open in the fluoride group following acid challenge.

**Conclusions:**

There is no significant difference between arginine and CSPS dentifrices in reducing dentin permeability following a single application and acid challenge. Following acid challenge, the reduced permeability generated by arginine and CSPS was more stable compared with the fluoride dentifrice.

## 1 INTRODUCTION

Dentin hypersensitivity is defined as a “short, sharp pain arising from exposed dentin in response to stimuli, typically thermal, evaporative, tactile, osmotic or chemical, which cannot be ascribed as arising from any other form of dental defect or disease” (Canadian Advisory Board on Dentin Hypersensitivity, 2003; Holland et al., 1997). The common causes of exposed dentin include gingival recession and enamel loss from improper tooth brushing or periodontal disease progression and its treatment (Addy, 2002; Canadian Advisory Board on Dentin Hypersensitivity, 2003; Markowitz & Pashley, 2008). Dentin hypersensitivity is a common complaint of individuals seeking dental treatment with a clinical prevalence ranging from 3%–57% (Addy, 2002; Cummins, 2010), whereas, the prevalence rises, ranging from 62.5%–90%, following non‐surgical periodontal therapy (Lin & Gillam, 2012). The hydrodynamic theory (Brännström, 1966) suggests that dentin hypersensitivity is caused by the outward flow of dentinal fluid in exposed tubules that subsequently activates intra‐pulpal nerve fibers (Addy, 2002; West, 2006). The greater number and wider diameter of open dentinal tubules observed in hypersensitive teeth suggest the possibility of elevated fluid flow that in turn increases dentin hypersensitivity (Absi et al., 1987, 1989).

Based on the physical dentin tubule occlusion approach for reducing dentin hypersensitivity, the use of bioactive glass, calcium sodium phosphosilicate (CSPS), for dentin remineralization was introduced in the mid‐1990s (Layer, 2011). When CSPS contacts saliva, it releases sodium ions that raise the local environmental pH that facilitates hydroxycarbonate crystal formation resulting from the release of calcium and phosphate ions (Andersson & Kangasniemi, 1991). Moreover, residual CSPS particles act as reservoirs that continuously release calcium and phosphate ions for several days (Damen & Ten Cate, 1992). An in‐vitro study found that a dentifrice containing CSPS created a hydroxyapatite‐like layer that sealed the dentinal tubules (Earl et al., 2011). Furthermore, a CSPS dentifrice also significantly reduced dentin permeability and created precipitates on the dentin surface, but was less resistant to acid challenge compared with either potassium nitrate or potassium oxalate dentifrices (Eliades et al., 2013; Wang et al., 2010). A clinical study revealed that a CSPS dentifrice was more effective compared with strontium‐based dentifrices in reducing dentin hypersensitivity after 6‐weeks (Du Min et al., 2008). In contrast, a clinical trial found that a CSPS dentifrice did not demonstrate a superior effect on reducing dentin hypersensitivity compared with a fluoride dentifrice (Zang et al., 2016). A meta‐analysis revealed that CSPS alleviated dentin sensitivity and post‐periodontal therapy sensitivity; however, the levels of evidence for these findings were classified as moderate and low, respectively (Zhu et al., 2015).

Arginine calcium carbonate (arginine) was also developed in the mid‐1990s for controlling dentin hypersensitivity (Cummins, 2010; Kleinberg, 2002). At physiologic pH, arginine and calcium, which are positively charged, bind to dentin surfaces and form a calcium‐rich layer to seal and block exposed tubules (Kleinberg, 2002). An in vitro study found that an arginine dentifrice blocked or narrowed dentinal tubules and was more stable over time compared with a strontium chloride dentifrice (Li et al., 2012). In addition, a dentifrice containing 8% arginine was more effective in reducing dentin permeability and tended to resist fruit juices and citric acid application compared with a strontium acetate dentifrice (Patel et al., 2011). Randomized clinical parallel‐design studies found that an arginine dentifrice provided hypersensitivity relief immediately and after a 3‐day use (Ayad et al., 2009; Nathoo et al., 2009). A meta‐analysis also showed a moderate benefit of arginine in alleviating dentin hypersensitivity compared with potassium, strontium, or fluoride dentifrices (Yang et al., 2016).

Currently, in vitro and clinical studies comparing the efficacy of CSPS and arginine on dentin hypersensitivity are limited. João‐Souza et al. (2018) reported that only CSPS paste and Gluma desensitizer, but not arginine, decreased the permeability post‐treatment following an erosive cycling. In contrast, an in vitro study from the same group showed that CSPS and arginine desensitizing dentifrices did not reduce dentin permeability during an erosive‐abrasive cycling model compared with anti‐erosive dentifrices (João‐Souza et al., 2019). Therefore, the aim of this study was to compare the efficacy of CSPS‐ or arginine‐containing dentifrices on dentin permeability and acid tolerance. The efficacy of these dentifrices in reducing permeability and tubule occlusion was evaluated using a modified dentin permeability assay and SEM, respectively.

## 2 MATERIALS AND METHODS

### 2.1 Dentin sample preparation

Extracted human third molars were collected following a protocol approved by the Human Research Ethics Committee of Chulalongkorn University. The teeth were thoroughly cleaned and stored in 0.1% thymol until used.

Dentin specimens were cut perpendicular to the long‐axis of the tooth above the cementoenamel junction to create 1‐mm thick dentin slices (discs) using a low‐speed water‐cooled diamond saw (Isomet®1000, Buehler Ltd., Lake Bluff, IL, USA). An average of 3 discs per tooth were obtained. Each dentin disc was examined to ensure that the specimen was free of coronal enamel and above the level of the pulp chamber.

### 2.2 Dentifrices

The test products comprised (1) a fluoride dentifrice (Colgate® Cavity Protection; Colgate‐Palmolive [Thailand] Company, Chonburi, Thailand), (2) a CSPS containing dentifrice (Sensodyne® Repair & Protect, GlaxoSmithKline, UK), and (3) an Arginine containing dentifrice (Colgate® Sensitive Pro‐relief™, Colgate‐Palmolive [Thailand] Company, Chonburi, Thailand).

### 2.3 Dentin permeability determination

#### 2.3.1 Dentin permeability determination following a single dentifrice application

Sixty dentin discs were soaked in 0.5 M EDTA for 24 h to remove the smear layer and smear plug. The maximum dentin permeability of each disc was determined. The permeability of each disc was assigned a value of 100%. The etched disc was rinsed and kept moist in artificial saliva until used in further experiments. The dentin discs were randomly assigned to three groups (*n* = 20). The discs were brushed with plain fluoride, CSPS, or arginine dentifrices for 1 min with an automatic toothbrush (Fresh‐Ex Battery toothbrush, Dr. Phillips®, Hong Kong). The permeability of each disc was re‐measured and the results are presented as the percentage of maximum EDTA‐etched permeability (Wang et al., 2010).

#### 2.3.2 Dentin permeability determination following acid challenge

After brushing, the dentin discs in each group were subjected to an acid challenge. Each disc was soaked in 6% citric acid (pH 1.5) for 1 min then washed with distilled water as previously described (Wang et al., 2010). The permeability of each dentin disc was then measured.

### 2.4 Permeability measurement

A modified dentin permeability measurement system was used as previously described (Wang et al., 2010). Briefly, a dentin disc was placed tightly between two “O” rings, which had a surface area of approximately 0.78 cm^2^ for water filtration. The upper “O” ring was covered with a glass slab to seal the system and the lower “O” ring was connected to a water‐filled system at 20‐cm H_2_O, which mimics the pulpal pressure. An air bubble was introduced into the water‐filled system using a syringe. The dentin permeability of each dentin disc was determined by measuring the time it took for the air bubble to move through a capillary tube. The time for the movement of the bubble was converted to hydraulic conductance (Lp) for each dentin disc, which was calculated using the following equation (Pashley et al., 1996):

$$\mathrm{Lp}=\mathrm{Jv}/A\left(P\right)$$

Jv; fluid flow (μl/min), A; dentin area for fluid filtration (cm^2^), and P; water pressure (cm H_2_O).

The percentages of dentin permeability (%Lp) were calculated. The mean percentage reduction between before, following brushing or acid challenge on the same disc was considered as the efficacy in reducing dentin permeability and acid tolerance.

### 2.5 SEM analysis

#### 2.5.1 Tubule occlusion following a single dentifrice application

Ten dentin discs were used to evaluate dentinal tubule occlusion. Each dentin disc was split into 4 pieces for four groups (1) No treatment, (2) brushed 1 min with fluoride, (3) CSPS, or (4) arginine dentifrices. The specimens were air‐dried at room temperature and coated with gold. The dentin disc morphology was examined and photographed using a scanning electron microscope (Quanta™ 250, FEI Company, Eindhoven, Netherland).

#### 2.5.2 Tubule occlusion following acid challenge

Five dentin discs per group were additionally prepared and the dentinal tubule occlusion was evaluated following acid challenge. Each disc was split into two pieces then brushed with fluoride, CSPS, or arginine dentifrice for 1 min. The two pieces were soaked in either 6% citric acid or artificial saliva for 1 min, then washed with distilled water. The specimens were air‐dried at room temperature and coated with gold. The tubule occlusion was examined and photographed using a scanning electron microscope.

### 2.6 Statistical analysis

The data were analyzed using SPSS (IBM Statistics version 22). Lp, %Lp, and the percentage change are presented as median (range). The Related‐samples Wilcoxon Signed Rank test was used to determine the differences between before and after treatment within each group. The non‐parametric Kruskal–Wallis test and post‐hoc (Mann–Whitney *U* test) were used to determine the differences in percentage reduction in dentin permeability between the three groups. A *p*‐value less than 0.05 was considered as significant. For the post‐hoc test, an adjusted *p*‐value less than 0.017 was considered significant.

## 3 RESULTS

### 3.1 Dentin permeability measurement

#### 3.1.1 The reduction of dentin permeability following a single dentifrice application

The decrease in hydraulic conductance (Lp) following brushing in all groups indicated reduced dentin permeability (Table 1). In addition, Lp following brushing with the dentifrices containing fluoride, CSPS, or arginine were significantly decreased within each group compared with the baseline (*p* < 0.0001).

**TABLE 1:** Hydraulic conductance (Lp) following brushing dentin discs with fluoride, CSPS, or arginine dentifrices

	Hydraulic conductance (Lp)		
	Fluoride	CSPS	Arginine
Before	0.17 (0.02–1.46)	0.14 (0.01–1.91)	0.18 (0.02–0.96)
After brushing	0.10* (0.01–0.93)	0.12* (0.01–0.95)	0.11* (0.01–0.50)

*Note*: The values are reported as median (range).

* Compared with baseline Lp values within each group (*p* = 0.0001).

The percentage reduction in dentin permeability after using the three different dentifrices was determined based on the 100% permeability of EDTA‐etched (Table 2). After a 1‐min brushing, the reduction in dentin permeability within each group was significant (*p* < 0.0001). Arginine demonstrated the highest reduction in dentin permeability (39.26%). The use of CSPS or fluoride dentifrices also reduced dentin permeability; however, the percentage of the reduction (32.27% and 21.71%, respectively) was inferior to that of the arginine group. Furthermore, comparing the 3 groups, only the arginine dentifrice was more effective in reducing permeability than fluoride (*p* < 0.011). CSPS reduced permeability better compared with the control; however, the difference was not significant (*p* = 0.056). Compared with arginine, the difference in dentin permeability reduction between the CSPS and fluoride groups was not significant (*p* = 0.398; Table 2).

**TABLE 2:** The percentage reduction in dentin permeability following brushing and acid challenge with three different dentifrices

	Percentage of dentin permeability (%LP)						
	Baseline	After brushing	% reduction after brushing	*p*‐value	Acid challenge	% increase after acid challenge	*p*‐value
Fluoride	100	78.29* (48.49–99.46)	21.71 (0.54–51.51)		96.45** (59.34–100)	11.99 (0.32–51.21)	
CSPS	100	67.73* (10.10–88.23)	32.27 (11.77–89.90)	0.056^a^	81.45** (44.41–99.36)	16.23 (0.38–58.51)	0.001^a^
Arginine	100	60.74* (19.17–91.78)	39.26 (8.22–80.83)	0.011^a^ 0.398^b^	70.15** (20.04–100)	5.53 (0–30.59)	0.0001^a^ 0.211^b^

*Note*: The percentages of dentin permeability are shown as median (range). The percentage at the baseline was considered the maximum permeability (100%).

^*^ Compared with baseline (*p* < 0.001).

** Compared with after brushing (*p* < 0.001).

^a^ Compared with fluoride.

^b^ Compared between the CSPS and arginine groups.

#### 3.1.2 The effect on dentin permeability following acid challenge

**FIGURE 1:**
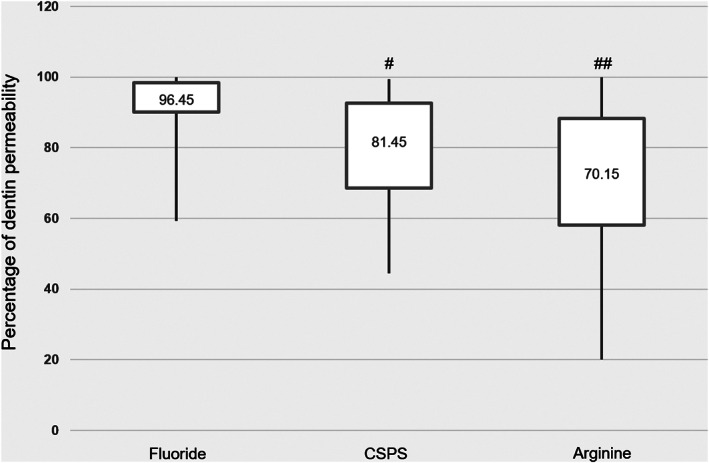
The efficacy of fluoride, CSPS and arginine dentifrices on acid tolerance. #, Compared between fluoride and CSPS groups (*p* = 0.001). ##, Compared between fluoride and arginine groups (*p* < 0.001)

The percentage of dentin permeability increased following acid challenge in all three groups (Table 2 and Figure 1). The dentin permeability after acid challenge in the CSPS and arginine groups remained lower than the control (*p* = 0.001, and *p* < 0.001, respectively). Although the arginine group had the highest potential benefit in acid tolerance, the efficacy of CSPS and arginine after acid challenge was statistically comparable (*p* = 0.211; Table 2 and Figure 1).

### 3.2 Dentinal tubule occlusion using SEM evaluation

#### 3.2.1 Dentinal tubule occlusion following a single dentifrice application

**FIGURE 2:**
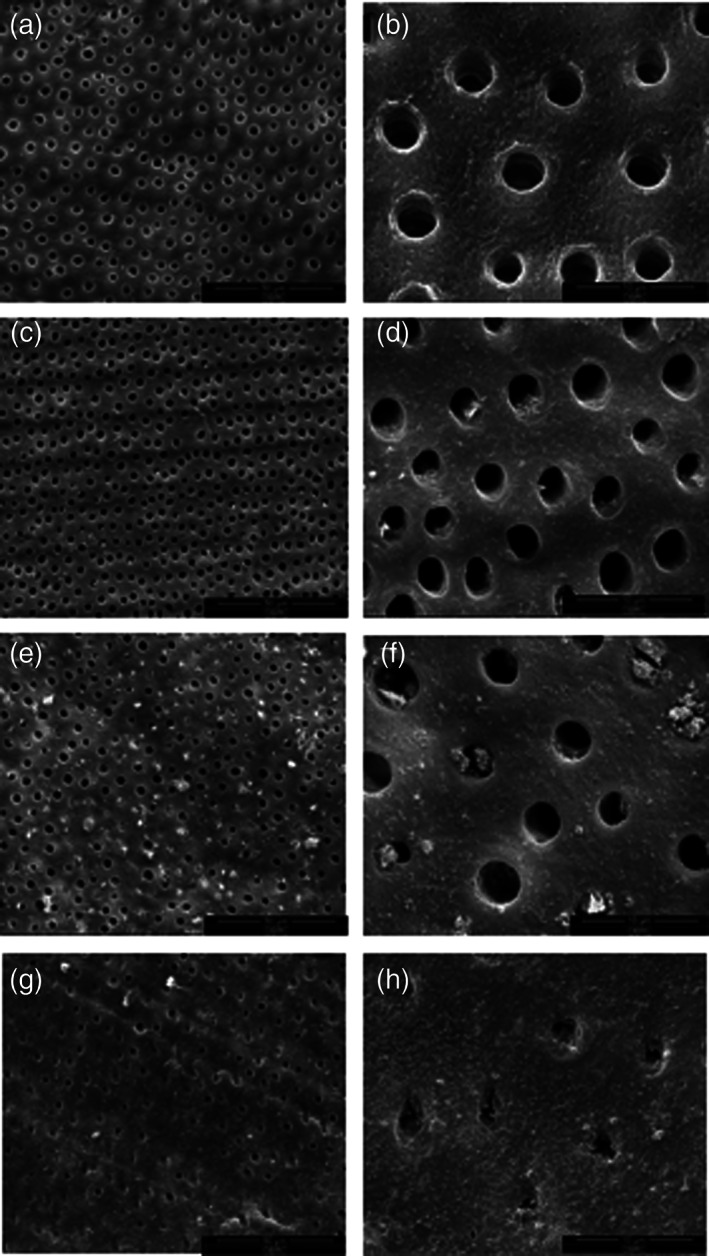
SEM images of the dentin surface morphology at 2000X (left panels; bar = 50 μm) and 10000X (right panels; bar = 10 μm). Dentin discs treated with EDTA (a), (b), then brushed with fluoride (c), (d), CSPS (e), (f), or arginine containing dentifrices (g), (h)

We obtained SEM images of the dentin surface morphology after the different treatments (Figure 2). The SEM images of the dentin surfaces indicated that the EDTA etching for 24 h removed the smear layer and smear plug from the dentin surfaces (Figure 2a). Most of the dentinal tubule orifices were completely open (Figure 2b). The dentin treated with the fluoride dentifrice exhibited fine debris on the dentin surfaces; however, most of the tubule orifices remained open (Figure 2c,d). In contrast, the dentin treated with the CSPS dentifrice showed a large amount of deposits on the dentin surfaces and within the dentinal tubule orifices (Figure 2e,f). The dentin treated with the arginine dentifrice also demonstrated a large amount of deposits on the dentin surfaces and within dentinal tubule orifices (Figure 2g,h). In addition, at a higher magnification, the complete occlusion of some tubules was observed in the arginine group (Figure 2h).

#### 3.2.2 Dentinal tubule occlusion following acid challenge

**FIGURE 3:**
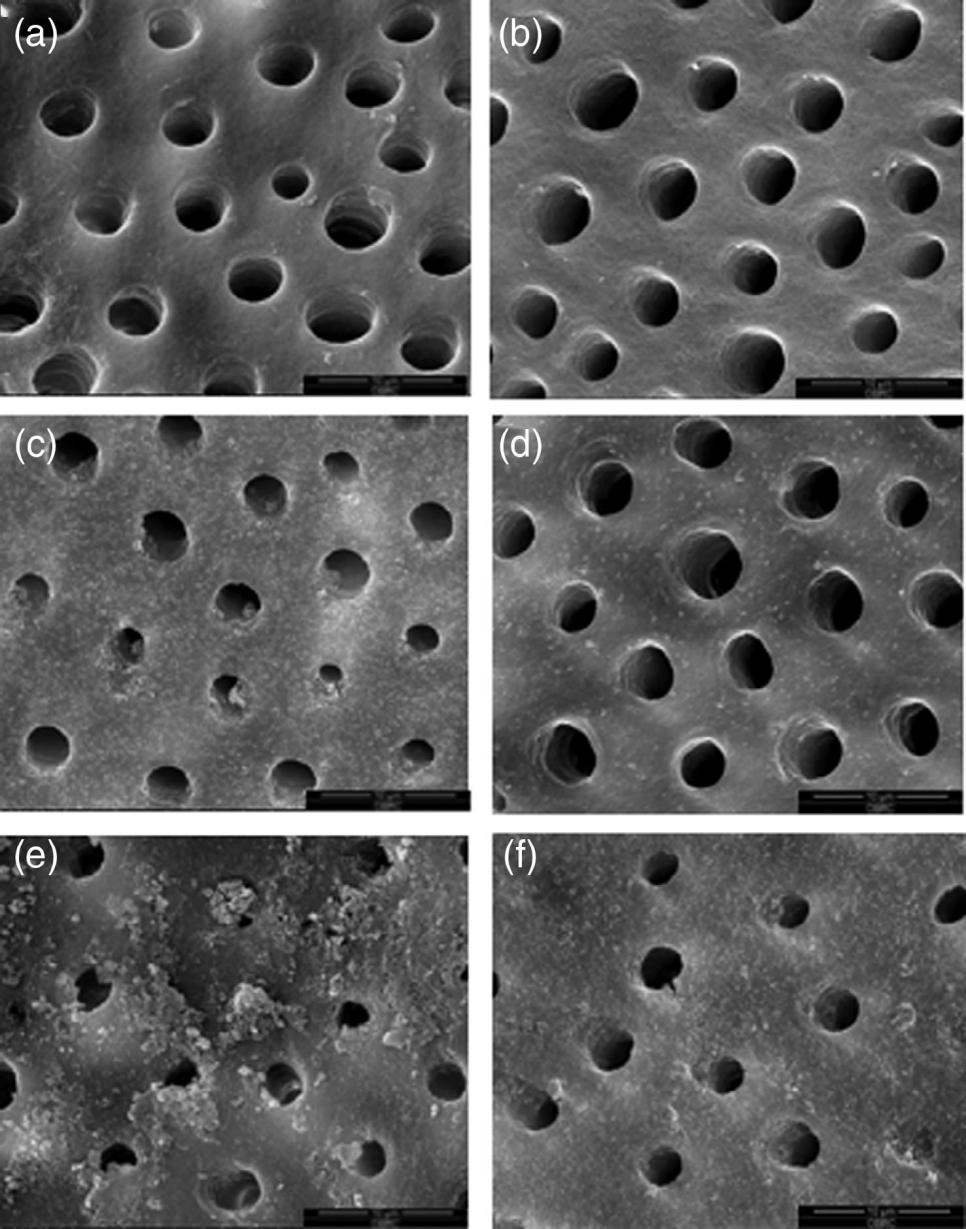
SEM images of dentin surface morphology following acid challenge at 10000X (bar = 10 μm). Each dentin disc was etched with EDTA then brushed with fluoride (a), (b), CSPS (c), (d), or arginine (e), (f) containing dentifrices. Half of each group were subjected to acid challenge (b), (d), (f)

The dentin treated with the fluoride dentifrice exhibited a small amount of debris in some tubule orifices (Figure 3a), and almost all of the orifices were open following citric acid application (Figure 3b). The CSPS group presented some remnants in the dentinal tubules (Figure 3c); however, some tubules reopened after acid challenge (Figure 3d). Furthermore, the dentin treated with the arginine dentifrice had a large amount of debris on the dentin surfaces and within the dentinal tubules (Figure 3e). Even though, precipitate remnants were observed, the tubule diameters were wider following acid challenge (Figure 3f).

## 4 DISCUSSION

Previous studies also found that a dentifrice containing arginine resulted in reduced dentin permeability (Banomyong et al., 2013; Patel et al., 2011; Pinto et al., 2014). CSPS prophylaxis paste or a dentifrice also generated reduced dentin permeability compared with the controls (Wang et al., 2010; Yilmaz et al., 2017). However, there is little information on the comparison of the efficacy of commercially available arginine or CSPS dentifrices in reducing dentin permeability.

The current study demonstrated that the arginine dentifrice reduced permeability more compared with the regular dentifrice, while CSPS had a slight benefit compared with the control. The dentin permeability or hydrodynamic conductance of the dentin surface is associated with the number and diameter of the dentinal tubules. In our setting, the number of tubules on each disc was relatively constant, thus, the changes in dentin permeability likely depends on the number of patent dentinal tubules and the degree of dentin occlusion after treatment.

To verify if the reduced permeability of the dentin disc was due to dentinal tubule blockage, the dentinal tubule occlusion was observed using SEM. Similar to previous studies (Earl et al., 2011; Li et al., 2012), our findings indicated that brushing with dentifrices containing CSPS or arginine resulted in dentinal tubule occlusion. Moreover, a single application of the CSPS or arginine dentifrice was more effective in blocking dentinal tubules compared with the fluoride dentifrice, which is in agreement with prior in vitro studies (Pinto et al., 2014; Wang et al., 2010).

Although there was no significant difference in permeability between the CSPS and arginine treatments, our results demonstrated that only arginine provided more benefit in permeability reduction compared with the control. This may be because complete tubule occlusion occurred after 24 h of CSPS application (Wang et al., 2010). However, Chen et al. (2015) demonstrated better dentin occlusion using CSPS prophylaxis paste compared with arginine based in‐office use.

Recently, an erosion abrasion cycling model demonstrated that desensitizing dentifrices containing arginine or CSPS did not decrease dentin permeability or induce dentin occlusion compared with anti‐erosive dentifrices (João‐Souza et al., 2019). In contrast, Lopes et al. (2018) showed that fewer opened tubules were only observed in the CSPS group following abrasion and erosion/abrasion cyclings  . These findings suggest that experimental design, exposure time, and other variable factors, such as the number of applications or application methods/settings may affect the amount of dentin occlusion.

To achieve sensitivity relief, the precipitates deposited following applying a desensitizing agent should resist daily acidic diet and drinks. Our finding indicated that the dentin occlusion created by CSPS or arginine dentifrices was more resistant to acid challenge than the fluoride dentifrice. Several studies also demonstrated that the precipitates from arginine or CSPS application prevented increased dentin permeability; however, the precipitates were partially lost after acid challenge, leading to slightly increased dentin permeability (Olley et al., 2015; Parkinson et al., 2010; Patel et al., 2011; Sharma et al., 2013; Wang et al., 2015; Yilmaz et al., 2017). Rajguru et al. (2017) revealed that arginine was slightly superior to CSPS in dentinal tubule occlusion, but demonstrated lower tolerance to acid challenge. In contrast, a study reported that CSPS paste reduced dentin permeability following a 5‐day erosion‐abrasion cycle compared with the arginine dentifrice (João‐Souza et al., 2018). Our results demonstrated that some precipitates formed by arginine or CSPS dentifrices were still present following acid challenge and might help in maintaining the reduced dentin permeability.

Because the present study was performed using only a single dentifrice application and one cycle of acid challenge, studies using repeated applications and more erosive cycles should be performed to mimic what occurs in the oral cavity over time. Moreover, the mineral ions in saliva also precipitate and occlude the tubules. This experiment was performed without saliva to observe the direct effect of desensitizers; therefore, the results possibly differ if saliva is taken into consideration.

The results of prior dentin occlusion and permeability following acid challenge studies were disparate and inconclusive due to differences in the number of applications, type of acid used, and experimental design. This study revealed only an immediate effect of the evaluated agents; therefore, further investigations focusing on the long‐term use of these products or clinical trials should be conducted. These studies would allow the establishment of appropriate protocols for using each product to best relieve dentin hypersensitivity.

## 5 CONCLUSIONS

There is no significant difference in the reduction in dentin permeability between arginine and CSPS dentifrices following a single application and acid challenge. Following acid challenge, the reduction in permeability generated by the arginine and CSPS dentifrices was more stable compared with the fluoride dentifrice.

## AUTHOR CONTRIBUTIONS

**Chantrakorn Champaiboon:** Conceptualization (lead); methodology (equal); funding acquisition; writing – original draft (lead); formal analysis (equal); writing – review and editing (lead).

**Attawood Lertpimonchai:** Methodology (equal); writing – original draft (supporting); formal analysis (equal); writing – review and editing (supporting).

**Kullanun Lertpimonchai:** Methodology (equal); writing – original draft (supporting); investigation; formal analysis (equal).

## CONFLICT OF INTEREST

The authors do not have any financial interest in the companies whose materials are included in the article.

## Data Availability

The data that support the findings of this study are available from the corresponding author upon reasonable request.
